# Cycling and bone health: a systematic review

**DOI:** 10.1186/1741-7015-10-168

**Published:** 2012-12-20

**Authors:** Hugo Olmedillas, Alejandro González-Agüero, Luis A Moreno, José A Casajus, Germán Vicente-Rodríguez

**Affiliations:** 1 GENUD 'Growth, Exercise, NUtrition and Development' Research Group, Universidad de Zaragoza, Zaragoza, Spain; 2 Faculty of Health and Sport Sciences (FCSD), Department of Physiatry and Nursing, Universidad de Zaragoza, Huesca, Spain; 3 School of Health Science (EUCS), Universidad de Zaragoza, Zaragoza, Spain

**Keywords:** cyclists, osteopenia, osteoporosis, sport, training

## Abstract

**Background:**

Cycling is considered to be a highly beneficial sport for significantly enhancing cardiovascular fitness in individuals, yet studies show little or no corresponding improvements in bone mass.

**Methods:**

A scientific literature search on studies discussing bone mass and bone metabolism in cyclists was performed to collect all relevant published material up to April 2012. Descriptive, cross-sectional, longitudinal and interventional studies were all reviewed. Inclusion criteria were met by 31 studies.

**Results:**

Heterogeneous studies in terms of gender, age, data source, group of comparison, cycling level or modality practiced among others factors showed minor but important differences in results. Despite some controversial results, it has been observed that adult road cyclists participating in regular training have low bone mineral density in key regions (for example, lumbar spine). Conversely, other types of cycling (such as mountain biking), or combination with other sports could reduce this unsafe effect. These results cannot yet be explained by differences in dietary patterns or endocrine factors.

**Conclusions:**

From our comprehensive survey of the current available literature it can be concluded that road cycling does not appear to confer any significant osteogenic benefit. The cause of this may be related to spending long hours in a weight-supported position on the bike in combination with the necessary enforced recovery time that involves a large amount of time sitting or lying supine, especially at the competitive level.

## Background

Participation in cycling has been shown to confer several health benefits in terms of improvements in cardiovascular fitness, reductions in mortality, and reduced cardiovascular risk factors as well as a reduced risk of cancer [1]. From a public health point of view, cycling is a widely practiced non-weight-bearing sport around the world, especially in Europe [2] and the bicycle is also used as a vehicle by millions of people in many different countries, accounting for an important part of their daily physical activity. However, as a non-weight-bearing activity, cycling practice is frequently associated with lower levels of bone mass [3]; in fact, two-thirds of the professional and master adult road cyclists could be classified as osteopenic [4].

Osteoporosis generally affects older populations and is characterized by 'low bone density and microarchitectural deterioration of bone tissue with a consequent increase in bone fragility and susceptibility to fracture' [5]. Low levels of bone mineral density (BMD) during earlier stages of life may contribute to the development of osteoporosis later on in life [6].

Many aspects of bones account for bone strength and resistance against fracture: bone mineral content (BMC), BMD, bone size, structure and microarchitecture, among others [7]. Genetic predisposition, dietary patterns, endocrine and environmental factors also play determining roles in bone health and maintenance throughout life [8,9]. Among the many environmental factors, physical activity and participation in sport promote health benefits in bone mass across all ages among different populations [10-14].

With regard to endocrine factors, the net product of bone formation and bone resorption, namely bone turnover, may be estimated by biomarkers involved with bone metabolism such as bone alkaline phosphatase (BAP), osteocalcin (OC) or C-terminal collagen crosslinks (CTX), which are among the most commonly studied in sport sciences [15].

This review aims to summarize the current available literature concerning bone mass and bone metabolism in cyclists, to observe whether the findings of the collective research are commensurate with the general idea that cycling has a deleterious effect on bone mass.

## Methods

### Search strategy

Journal articles were identified from MedLine (1965 to April 2012), EMBASE, Web of Science and SportDiscus. The search strategy used to identify the articles was: '((bone) or (bmc) or (bmd) or (osteoporosis)) AND ((cyclist) or (cyclists) or (cycling sport) or (triathlon) or (triathletes))' to identify the articles on the topic of this review. Additional articles were added after reviewing the references of the previous researches. This produced a total of 214 citations. MOOSE [16] and PRISMA [17] guidelines for the reporting of systematic reviews were followed for observational and interventional studies respectively.

### Inclusion criteria

The inclusion criteria for this review were: (a) bone mass or bone metabolism markers, and not fractures or any other topic, had to be the main subject of each study; (b) the studies had to include cyclists or triathletes and not only athletes or sportspeople; (c) the type of studies to be included had to be original research manuscripts (cross-sectional, interventional, follow-up or retrospective studies); reviews or published abstracts were not included; (d) only papers written entirely in English were included in this review.

### Exclusion criteria

**Figure 1:**
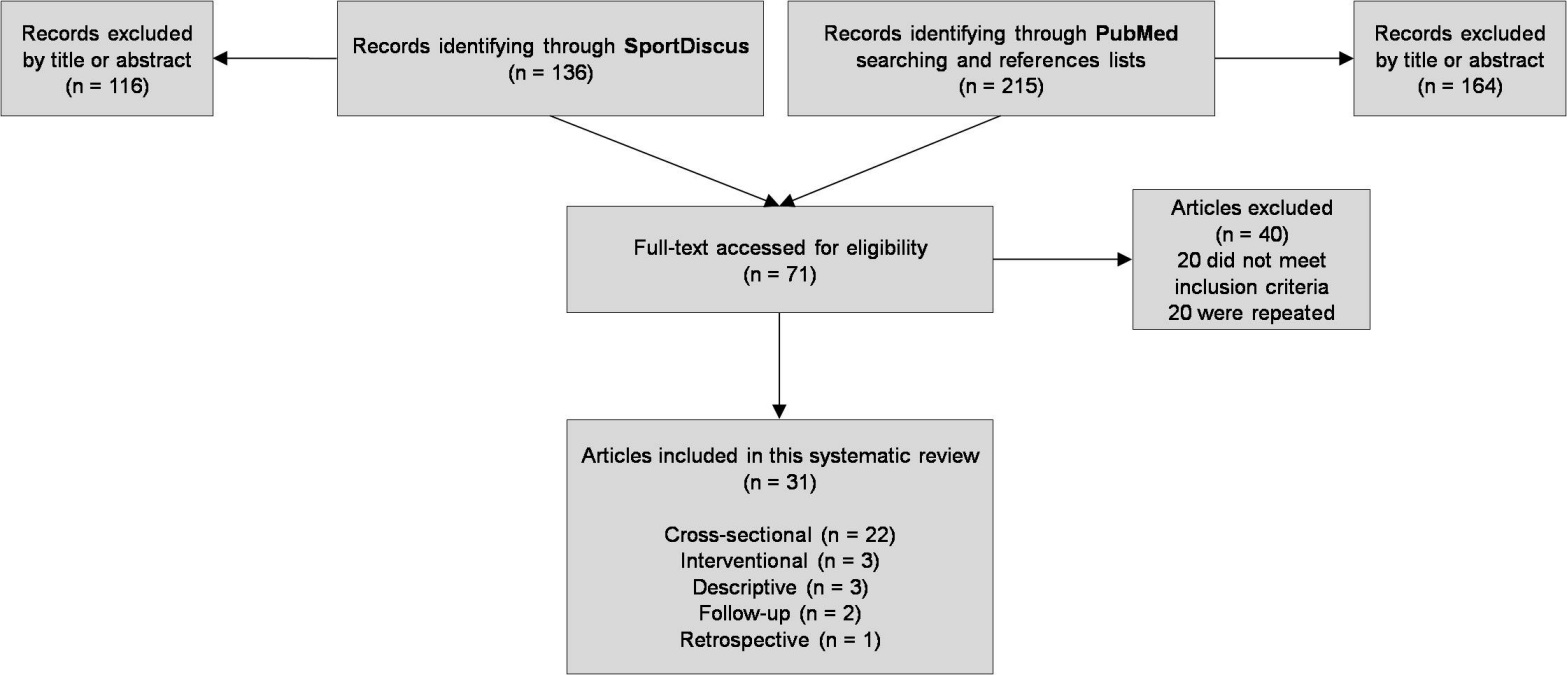
**Flow chart diagram of the study selection process**.

The inclusion criteria were applied to the 215 citations by 2 authors independently; if disagreement occurred, all authors reviewed the data until consensus was achieved. Of the total of 215 citations, 31 references fulfilled the inclusion criteria (Figure 1). A total of 184 citations were excluded for the following reasons: 145 did not include bone mass or bone metabolism markers as their main topic, 17 were not original articles and 22 were not written in English.

### Data extraction

Each study was independently evaluated. General information about the article title and type of the study, author(s), characteristics of the participants (age, sex, and exercise), comparison group(s) (if available), data source and results were also extracted.

Table 1 summarizes all the studies concerning bone mass and cycling participation included in this review. Different age stages (adolescents, adults, older adults), genders, competitive levels, years of practice and groups of comparison (that is, sedentary controls, runners, and so on) were included in this review, making the comparison between studies rather difficult; however, efforts have been made to summarize and clarify the current knowledge on this topic despite discrepancies in these variables.

**Table 1:** Studies concerning bone mass, bone metabolism and cycling participation

Study	Participants			Exercise	Years of cycling training	Study design	Data source	Results^a^	Strength of evidence
							
	Number	Sex	Age						
Barry *et al. *(2007) [47]	CYC (20)	M	22 to 45	Competitive level		2-h exercise bout at 60% to 75% VT	Hormones, calcium	Parathyroid was increased after 2 h of cycling.	B: observational
Barry *et al. *(2008) [43]	CYC (14)	M	27 to 44	>450 h/year	4.9 ± 2.4	Two groups: HIGH and LOW calcium supplementation over 1-year season	DXA	Both groups decreased BMD over 1 year in total hip and subregions, without differences for HIGH or LOW calcium.	A: RCT
Barry *et al. *(2011) [39]	CYC and TRI (20)	M	37 ± 7.6	-	6.0 ± 6.5	Different calcium supplementation groups over three 35-km trials	DXA, hormones	30% of participants had LS BMD T-score over -1.0. Calcium supplementation attenuates disruption of parathyroid hormone.	A: RCT
Beshgetoor *et al. *(2000) [41]	CYC (12); RUN (9); CON (9)	F	49.6 ± 7.9	-	-	18 months follow-up	DXA, calcium intake	Femur BMD maintained in CYC and RUN, decline CON. LS BMD maintained RUN, decline CYC and CON. No relationship between BMD and calcium intake.	B: case-control
Brown *et al. *(2000) [49]	CYC (32)	M/F	16 to 62	Competitive cycling	>2	Two groups: HIGH FAT and HIGH CARBOHYDRATE intake; 12-week intervention	DXA	No differences in fat or lean accumulation between groups. BMD increased in HIGH FAT group.	A: RCT
Campion *et al. *(2010) [34]	CYC (30) CON (30)	M	29 ± 3.4 28 ± 4.5	22 to 25 h/week <1 h/week	-	Cross-sectional	DXA	CYC lower WB, LS, pelvis, femoral neck, upper and lower limbs than CON	B: case-control
Duncan *et al. *(2002) [21]	CYC (15) RUN (15) SWI (15) TRI (15) CON (15)	F	16 to 17	≥8 h/week ≥8 h/week ≥8 h/week ≥8 h/week <2 h/week	3.1 ± 1.8	Cross-sectional	DXA	CYC lower legs BMD than RUN. No differences with CON.	B: case-control
Duncan *et al. *(2002) [45]	CYC (10) RUN (10) SWI (10) TRI (10) CON (10)	F	16 to 17	≥8 h/week ≥8 h/week ≥8 h/week ≥8 h/week <2 h/week	3.1 ± 1.8	Cross-sectional	MRI, DXA	CYC lower cortical CSA, moment of inertia and mid-femur BMD than RUN. No differences with CON.	B: case-control
Fiore *et al. *(1996) [36]	CYC (14); CAN (18); CON (28)	M	-	-	-	Cross-sectional	DXA	CYC lower WB, LS and pelvic BMD than CAN. No differences with CON.	B: case-control
Guillaume *et al. *(2012) [38]	CYC (29)	M	26.5 ± 5.3	25,000 to 30,000 km/year	4.5 ± 4	Descriptive	DXA, bone markers	CYC lower LS BMD Z-scores. Bone turnover markers were in a normal range.	B: case series
Heinonen *et al. *(1993) [19]	CYC (22); ORI (30); SKI (28); CYC (29); WL (18); CON (25)	F	18 to 32	-	-	Cross-sectional	DXA, calcium intake	CYC lower BMD at all sites than WL. No differences with CON. No relationship between BMD and calcium intake.	B: case-control
Hinrichs *et al. *(2010) [35]	CYC (16) RUN (37) TRI (22) TEAM (62) POW (45) BAL (13) STU (126) CON (61)	M/F	17 to 30	15 h/week 12.5 h/week 15 h/week 10 h/week 10 h/week 27 h/week 7.5 h/week -	>4	Cross-sectional	DXA	CYC low values of LS and femur BMD than the other groups	B: case-control
Maïmoun *et al. *(2003) [25]	CYC (11) SWI (13) TRI (14) CON (10)	M	18 to 39	10.6 h/week 10.7 h/week 15.2 h/week <2 h/week	-	Cross-sectional	DXA, hormones	CYC and TRI induce androgen deficiency compared to CON, without alteration in BMD	B: case-control
Maïmoun *et al. *(2004) [24]	CYC (11) SWI (13) TRI (14) CON (10)	M	18 to 39	10.6 h/week 10.7 h/week 15.2 h/week <2 h/week	9.3 ± 6.8	Cross-sectional	DXA, bone markers, calcium intake	CYC lower BAP than all groups. No differences in BMD. No relationship between BMD and calcium intake.	B: case-control
Medelli *et al. *(2009) [29]	CYC (73) CON (30)	M	25.8 ± 4.3 28.3 ± 4.5	≥3 to 6 h/day <1 h/week	-	Cross-sectional	DXA, calcium intake	CYC had higher calcium intake and lower LS and femoral neck BMD than CON.	B: case-control
Medelli *et al. *(2009) [4]	CYC (23)	M	28.5 ± 3.9	≥3 to 6 h/day	-	Descriptive	DXA	Two-thirds of participants had lower values of LS BMD	B: case series
Morel *et al. *(2001) [27]	CYC (47); other sports (657)	M	30	7 h/week	-	Cross-sectional. Sportsmen classed as amateur when 11 to 18 years old.	DXA	No differences between different sports	B: case-control, retrospective
Nevill *et al. *(2004) [23]	CYC (16) CON (15) Others (90)	M	28.6 ± 6 24.9 ± 5.4 -	>4 h/week - -	>3	Cross-sectional	DXA	CYC had no differences in BMD compared to CON, as other sports do	B: case-control
Nichols *et al. *(2003) [32]	Young CYC (16) Master CYC (27) CON (24)	M	31.7 ± 3.5 51.2 ± 5.3 51.2 ± 2	≥10 h/week ≥10 h/week <2 days/week	10.9 ± 3.2 20.2 ± 8.4 -	Cross-sectional	DXA	Master CYC lower WB BMD than young CYC. Master CYC lower LS and hip BMD than young CYC and CON.	B: case-control
Nichols *et al. *(2010) [42]	CYC (19) CON (18)	M	50.7 ± 4 50.7 ± 4.1	11.1 h/week 4.5 h/week	27.5 ± 6.8	Longitudinal, 7-year follow-up	DXA	Higher percentage of CYC osteopenic/osteoporotic than CON. Greater increment in this percentage in CYC.	B: case-control
Nikander *et al. *(2005) [26]	CYC (29) SWI (27) VOL (21) HUR (24) SQU (20) SOC (19) SKA (15) AER (27) WL (19) ORI (29) CRO (25) CON (30)	F	20 to 30	10.2 ± 6.8 13.5 ± 4.5 9.9 ± 2.5 9.1 ± 2.4 6.0 ± 3.1 8.6 ± 5.5 6.4 ± 3.6 6.6 ± 3.7 8.3 ± 2.6 8.6 ± 1.4 10.9 ± 1.2 2.9 ± 2.0	5.9 ± 3.1 10.6 ± 4.3 8.6 ± 3.3 10.4 ± 3.0 6.4 ± 4.7 10.7 ± 3.8 9.4 ± 7.2 8.3 ± 2.7 3.3 ± 1.3 13.0 ± 3.1 10.7 ± 3.5 -	Cross-sectional	DXA, calcium intake	CYC and SWI no differences with CON in BMD and CSA, as the rest of the sports. No differences in calcium intake.	B: case-control
Olmedillas *et al. *(2011) [40]	CYC (21) CON (23)	M	15 to 21	10 h/week 4 h/week	2 to 7	Cross-sectional	DXA	CYC lower BMC at WB, pelvis, FN and legs, and lower BMD at pelvis, hip and legs. Greater differences in CYC over 17 years compared to CON.	B: case-control
Penteado *et al. *(2001) [22]	CYC (31) CON (28)	M	24 26	21 h/week 0	5.2 ± 3.3	Cross-sectional	DXA	No differences with CON.	B: case-control
Rector *et al. *(2008) [37]	CYC (27) RUN (16)	M	20 to 39	≥6 h/week ≥6 h/week	>2	Cross-sectional	DXA, bone markers	CYC lower WB and LS BMD, and 7 times more likely to have osteopenia than RUN. No differences in bone turnover markers.	B: case-control
Rico *et al. *(1993) [20]	CYC (22) CON (27)	M	16	≥10 h/week -	>2	Cross-sectional	DXA, calcium intake	CYC lower legs BMC than CON, without adjustment. No differences when adjusting by weight. No relationship between BMD and calcium intake.	B: case-control
Rico *et al. *(1993) [50]	CYC (22) CON (27)	M	16	≥10 h/week -	>2	Cross-sectional	DXA	CYC lower WB BMC and BMD than CON	B: case-control
Sabo *et al. *(1996) [30]	CYC (6) WL (28) BOX (6) CON (21)	M	21 to 24	3,000 to 10,000 km in pre-competition	-	Cross-sectional	DXA	CYC lower LS BMD than CON	B: case-control
Stewart *et al. *(2000) [31]	CYC (14) RUN (12) RUN+CYC (13) CON (23)	M	18 to 43	8.7 h/week 10.7 h/week 9.4 h/week 0 h/week	>2	Cross-sectional	DXA	CYC lower LS BMD than CON. RUN higher WB BMD than CON. RUN+CYC higher WB BMD than CON.	B: case-control
Smathers *et al. *(2009) [33]	CYC (32) CON (30)	M	20 to 45	≥1 year 3 days/week	9.4 ± 1.1	Cross-sectional	DXA, calcium intake, hormones	CYC higher calcium intake. No differences for testosterone. CYC lower LS BMD than CON.	B: case-control
Warner *et al. *(2002) [28]	Cross-country CYC (16) Road CYC (14) CON (15)	M	20 to 40	≥10 h/week ≥10 h/week <2 h/week	5.9 ± 2.8 9.9 ± 4.4 -	Cross-sectional	DXA, hormones	Cross-country CYC higher BMD at all sites that road CYC and CON. No differences in testosterone levels.	B: case-control
Wilks *et al. *(2009) [46]	Sprint CYC (52) Distance CYC (19) CON (32)	M/F	30 to 82 50 ± 13	<2 h/week	26 ± 15 29 ± 16 Start age	Cross-sectional	pQCT	Sprint CYC higher index of strength in tibia and radius than CON. Distance CYC higher tibial BMC than CON.	B: case-control

^a^Unless stated, the results indicate significant differences between two or more groups.

AER = step aerobicists; BAP = bone alkaline phosphatase; BMC = bone mineral content; BMD = bone mineral density; BOX = boxers; CAN = canoeists; CIC = cyclists; CON = controls; CRO = cross-country skiers; CTX = C-terminal collagen crosslinks; DXA = dual energy X-ray absorptiometry; HUR = hurdlers; LS = lumbar spine; MRI = magnetic resonance imaging; OC = osteocalcin; ORI = orienteers; pQCT = peripheral quantitative computed tomography; RCT = randomized control trial; RUN = runners; SKA = speed skaters; SKI = skiers; SQU = squash players; SWI = swimmers; TRI = triathletes; VOL = volleyball players; WB = whole body; WL = weightlifters.

### Strength of evidence

The guidelines of Hadorn *et al. *[18] were used to rate the quality of evidence of every study and the qualifications were also stated in Table 1. This method includes three levels of quality, as outlined below.

Level A: well conducted randomized control trials (RCT) with 100 participants or more (including multicenter and meta-analyses); well conducted RCT with fewer than 100 participants (one or more institutions and meta-analysis; well conducted study).

Level B: well conducted case-control study, poorly controlled or uncontrolled (including RCT with one or more major or three or more minor methodological flaws), observations studies with high potential for bias (case series with comparison to historical controls), case series or case reports, conflicting evidence with more support.

#### Level C: expert opinion

Additional file 1 evaluates the quality and strength of evidence of each study, providing a final score based on several objective questions.

## Results and discussion

### Cycling and bone mass

#### BMC and BMD

The vast majority of the reviewed studies used dual energy X-ray absorptiometry (DXA) devices for their investigations; in fact only 2 out of 31 studies did not include any DXA measurement in their results.

Heinonen *et al*., in 1993, first described and compared bone mass of young adult female cyclists with other different sportswomen and controls. They found that cyclists had lower BMD at all body sites than weightlifters, but no differences were observed with other sports or with controls [19]. Subsequent studies reinforced the finding that no differences in BMC or BMD were observed between cyclists and controls, in both males and females, either in adolescents [20,21] or adults [22-26], or compared with other competitive sportspeople [27]. Furthermore, Warner *et al. *found that adult cross-country cyclists had higher BMD than road cyclists and controls [28].

Despite the above-mentioned studies, the available literature largely describes lower levels of BMD and BMC for different bodily regions in cyclist participants almost at all ages and in both genders. The lumbar spine has probably been the most studied region in this regard, with fairly conclusive results that lower BMD can be observed in cyclists compared with controls [29-35], practitioners of other sports [19,35-37] or reference values [4,38,39]. Moreover, the pelvic and hip regions, as key areas for osteoporotic fractures, and the whole body have been studied with concerning results. Lower values of pelvis, hip and femoral neck BMD in male cyclists were found compared with controls at all ages [29,32,34,40], with higher differences in the older cyclists.

Longitudinal studies may help to understand whether the observed low bone mass is acquired during a period of time and then maintained, or whether in fact, cycling training has a harmful effect on this tissue. In this regard, Beshgetoor *et al. *showed that 18 months of training in competitive female cyclist resulted in maintenance of the femur BMD, but a declination in lumbar spine BMD [41]. More recently, Nichols *et al. *showed that not only were more master cyclists classified as osteoporotic compared to age-matched and weight-matched non-athletes, but also the percentage of osteoporotic cyclists increased significantly over a 7-year period [42]. Also, Barry *et al. *studied two groups of adult cyclists supplementing them with high and low calcium intakes; they observed that the declination in hip BMD was the same in both groups [43].

Most of the studies reviewed showed that cycling does not appear to have a beneficial effect on BMD, especially at the lumbar spine, with some cases showing detrimental effects compared to other osteogenic sports. Very few studies have been conducted on child or adolescent cyclists, but the available manuscripts allow us to hypothesize that the differences in BMD become greater with age. Also, longitudinal studies point to this by showing declinations even with calcium supplementation.

#### Bone geometry and structure

The DXA device creates a two-dimensional image of the bone that does not provide information about volumetric BMD (vBMD) and does not differentiate cortical and trabecular bone. Osteoporosis is highly related to BMD; however, strength indexes and, therefore, the risk of fracture have a close relationship with structural aspects of bones such as cortical thickness and bone cross-sectional area, among others [44]. Peripheral quantitative computed tomography (pQCT) and magnetic resonance imaging (MRI) are alternative bone densitometry techniques that allow separate evaluation of those bone regions. These techniques are also able to assess actual vBMD at peripheral sites as well as estimating geometric properties of bone that are related to bone strength, going beyond the scope of current DXA determinations.

Two studies of this nature performed on cyclists were identified. Using MRI, Duncan *et al. *found that female adolescent cyclists had lower bone cross-sectional area, moment of inertia and mid-femur vBMD than their runner counterparts, while no differences were observed with controls [45]. Wilks *et al. *compared pQCT values of male distance-trained and sprint-trained master cyclists (aged 30 to 82 years) with age-matched sedentary controls, finding that distance-trained athletes presented higher values at the tibial sites, and sprinters at both tibial and radial sites, than controls [46].

Due to the abundance of factors contributing to bone strength, it seems that, regardless of the lower values of BMD observed in studies with DXA, cycling does not negatively affect the geometry and/or structure of the bones measured with pQCT. What appears to be clear is that more in-depth studies are required in order to corroborate these findings, and to evaluate possible changes over different periods of time and life stages in these variables.

### Endocrine factors related with bone mass

#### Bone turnover markers

Bone turnover markers related to bone formation, such as BAP or OC and also CTX related to bone resorption, have been barely investigated in cyclist populations.

Rector *et al. *showed no differences between adult cyclists and runners in bone metabolism markers [37], and Guillaume *et al. *reinforced that by showing these markers in normal ranges in a group of young cyclists [38]. However, Maïmoun *et al. *[24] studied three groups of adult athletes (cyclists, triathletes and swimmers), compared to controls, and showed lower BAP in cyclists compared to any other group. No differences were found for OC or CTX among the three groups of athletes.

#### Hormonal profile

Smathers *et al. *and Warner *et al. *showed no differences in testosterone levels between adult male cyclists and controls [28,33]; however, Maïmoun *et al. *showed lower testosterone levels in adult male cyclists and triathletes compared with controls [25]. Two different interventional studies focused on the effects of cycling on parathyroid hormone concluded that it increased after 2 h of cycling [47], and that calcium supplementation attenuated its disruption [39].

Inconclusive results can be obtained from the few heterogeneous studies available on neuroendocrine factors affecting bone mass in cyclists; nevertheless, it may be stated that the hormonal status seems to be within normal ranges in this specific population and that the formation of bone appears to be somewhat reduced in a group of adult cyclists with a long history of training (over 9 years on average). For that reason, as bone metabolism does not seem to be the main factor regarding the general low mineral accumulation in cyclists' bones, other mechanical factors such as the lack of osteogenic impacts might also have an influence.

### Factors affecting bone mass

#### Diet and calcium intake

Some authors found that cyclists take in a higher amount of calcium than controls [29,33], but as it is believed that cyclists have a higher energy intake than controls due to higher energy expenditure [48], total energy intake is a factor that must be taken into account. However, no associations were found between calcium intake and BMD for adults and young adult cyclists [19,20,24,41]. In addition, Barry *et al. *found that BMD was decreased in a group of cyclists independently of their high or low calcium intake over a 1-year season [43], and that acute calcium supplementation decreased the parathyroid hormone disruption after cycling [39]. Werner *et al. *did not find differences in calcium or vitamin D intake between road and mountain cyclists and controls [28]. Brown *et al. *showed that a fat-rich diet yielded higher increments in BMD in cyclists than other carbohydrate-rich diets without variation in fat or lean masses over a period of 12 weeks [49].

Studies appear to indicate that there are no observable effects in consuming higher amounts of calcium to reduce the supposed detrimental effect of cycling on bone mass. Furthermore, from these data it is difficult to define whether or not cyclists do in fact require any additional calcium supplements. Further studies are necessary in order to confirm previous results and to elucidate whether diet may be a more important factor for cyclists during years of growth in comparison to athletes in other disciplines.

#### Age

As previously stated, adult cyclists showed lower BMD at several sites of the body (mainly then lumbar spine) compared to controls or practitioners of other sports; however, few of the included studies incorporated adolescents within their samples. These studies showed similar results in that, under the age of 18, the differences in BMD between cyclists and controls were not observable [40,45,50], as observed in adolescent runners [21]. In addition, Maïmoun *et al. *[24] stated that age, among other factors, could influence BMD in people that practice sports including cycling. Though using a different measurement technique (that is, pQCT) Wilks *et al. *[46] suggested that cycling activity could prevent bone strength losses in older people.

The positive effect that general sport practice has on bone is well known [10], and that maximizing bone mineral mass during growth may help to prevent fractures during adolescence and at an older age [51]. We have observed that cycling during the early years of life does not negatively affect the bones, yet it does not exert as much of a positive influence as other sports clearly do. Consequently, the age factor for cycling must be taken into account.

#### Gender

It is widely known that women have lower bone mass than men throughout life and that are at higher risk of suffering from osteoporosis; therefore it is important to ascertain whether gender has any influential effect on the accumulation of bone mass in cyclists.

The studies included in this review incorporating only women within their samples showed similar results in bone mass to studies on men or both genders when they are compared with other sportswomen or controls [19,21,41,45].

Therefore, because females are more likely to become osteoporotic, pre-participation examinations (that is, DXA scans) should be conducted in adult female cyclists training at a high level.

#### Training level and type of cycling practice

Few studies have been performed in low-level cyclist populations; Morel *et al. *studied amateur sportsmen without finding differences in bone mass between cycling and other sports [27]. In general, studies included in this review had samples of adult cyclists with a high level of training; however, some of them were performed in amateur or adolescent cyclists, showing that in fact the level of practice and/or the years of training might increase the risk of low bone mass.

The two studies by Duncan *et al. *showed no differences in BMD between cyclists and controls, but it is worth noting the sample of adolescents that they used [21,45]. The study by Wilks *et al*., which is discussed below, showed greater bone strength surrogates in cyclists compared to controls in a sample of adults and older adults with a high level of cycling practice during their adolescence and adulthood.

Cycling is a widespread activity that involves different disciplines (that is, mountain biking, road cyclists, BMX, and so on) and, in combination with swimming and/or running, forms part of triathlon or duathlon events.

What has been observed regarding combination of cycling with running is that this practice counteracts the effect that cycling has on bone mass by an increased total body BMD compared with controls not observed in the cycling alone group [31]. In triathletes Maïmoun *et al. *demonstrated a similar behavior to cyclists in terms of testosterone deficiency without differences in BMD [25]. Concerning different types of cycling, as expected, Wilks *et al. *demonstrated that sprint-trained cyclists had stronger bones than those training for longer distances [46], and Warner *et al. *showed that cross-country cycling practitioners acquire higher BMD than road cyclists [28].

From a review of the current available literature described herein it can be concluded that road cycling at a competitive level might be more detrimental for bone health than other forms of cycling such as mountain biking in a recreational way. However, it should be mentioned that an upper threshold of training level may exist which protects bones from fractures, perhaps by improving their geometry and/or structure. Moreover, and especially where elite cyclists are concerned, it is also possible that resistance training also provides significant positive influences on bone mass. It is noteworthy that duathlon and triathlon do not have the same harmful effect that cycling alone seems to have on bone mass.

## Limitations

Different sources of bias inherent to systematic reviews should be addressed. First, we excluded non-English publications; thus a possible language bias is inherent.

Second, the studies included in our systematic review were too heterogeneous to perform a meta-analysis. The lack of this type of analysis makes difficult to reach strong conclusions. However, Table 1 contains quantitative information on each individual study.

Third, we classified articles into categories based on their assessment method, and afterwards we discussed some factors affecting bone mass. We believe that the results are more easily read and understood with this categorization. Although we systematically assessed articles before assigning them into categories, categorization is not a closed issue.

## Conclusions

To date, a considerable number of studies have reported on the possible harmful effect of cycling on bone health; however there are still some pending issues that need to be addressed. In general, cycling participation seems to have a neutral effect on bone health in terms of low BMD at several sites of the body; however, some factors need to be taken into account regarding this assumption. Road endurance cycling at a professional level could be more detrimental to bone mass than performing this activity recreationally, or worse than performing other disciplines such as cross-country cycling or combinations of cycling with running [28]. In addition, there is some evidence that the practice of this sport during adolescence and adulthood could help to maintain a better bone geometry and strength later in life [46]. Few studies described bone mass in young cyclist populations, however, it can be hypothesized that the differences in BMD between cyclists and controls or other sport practitioners become greater from 17 years of age onwards [40]. It is important to note that, contrary to other sports, there have been no reports of any positive effects of cycling on bone mass during adolescence.

Factors affecting bone mass such as calcium intake and hormonal profile were found to be within the regular range in cyclists [28,29,33]; allowing us to believe that the lack of impact might be one of the main reasons for the low bone mass acquisition in this population. It is also thought that professional cyclists spend several hours daily resting after training time, and sedentary time has been associated with low bone mass [52]; this factor should be taken into account as it may also partially explain the low BMD observed in these athletes.

In general it can be concluded that individuals who practice non-weight-bearing sports such as cycling are more likely to develop osteopenia or osteoporosis [53]; and the high incidence of falls while cycling makes this fact even more relevant for this population [42,54].

In conclusion, road cycling at a competitive level is less effective at improving bone mass when compared with weight-bearing sports. Based on the available evidence, in general, cycling as a sole form of exercise is not recommended for people who are at risk of developing osteoporosis, unless it is complemented with some kind of osteogenic training.

### Practical recommendations and future research

The likelihood of a cyclist to develop osteopenia or osteoporosis at clinical sites due to low levels of BMD must be taken into account by health organizations, federations, trainers and athletes. In accordance with Maïmoun *et al. *there may exist a threshold, since BMD measurements are inadequate to detect slight and acute changes in bone metabolism [55], and therefore further research on bone metabolism, bone strength and structure in cyclists at different ages, based on previous studies [46], could help to better understand the actual bone weakness in cyclists. Also, longitudinal studies aimed at identifying whether the key periods of bone mass acquisition are affected by cycling are needed.

Secondly, Beatty *et al. *[56] and Nichols *et al. *[42] proposed to incorporate impact activities in training programs for cyclists, as it has been demonstrated that plyometric jumps increase bone mass in different populations [14,57]. Generally, amateur cyclists are unwilling to include resistance and/or plyometric exercise in their routine throughout the season despite there being no scientific evidence of performance impairment. Therefore, studies on the effects of different training implementations will define whether these interventions are enough to counteract the effect of cycling.

## Competing interests

The authors declare that they have no competing interests.

## Authors' contributions

All authors made substantive intellectual contributions to the final manuscript, and read and approved it.

## Pre-publication history

The pre-publication history for this paper can be accessed here:

http://www.biomedcentral.com/1741-7015/10/168/prepub

## Supplementary Material

Additional file 1 **Quality assessment tool of the included studies**. Additional file 1 provides measurement regarding the quality and strength of the evidence of each study included in the review. Click here for file
