# Supplementary material for: Cycling and bone health: a systematic review
Source: BMC Med. 2012 Dec 20;10:168. doi: 10.1186/1741-7015-10-168 (PMC3554602; doi:10.1186/1741-7015-10-168)
Supplement: Additional file 1 — Quality assessment tool of the included studies. Additional file 1 provides measurement regarding the quality and strength of the evidence of each study included in the review. [file 1741-7015-10-168-S1.DOCX]

**Additional file 1. Quality assessment tool of the included studies.**

| **Author and year** | **Appropriate Research Design?** | **Appropriate Recruitment Strategy?** | **Response Rate?** | **Is Sample Representative? (All clinic populations)** | **Objective and Reliable Measures?** | **Power Calculation/ Justification of Numbers?** | **Appropriate Statistical Analysis?** | **Evidence of Bias?** | **Quality Indicators Met** |
| --- | --- | --- | --- | --- | --- | --- | --- | --- | --- |
| Barry et al. (2007)[47] | Yes | Yes | Not reported | Unclear | Yes | No | Yes | No. | 4/7 |
| Barry et al. (2008)[43] | Yes | Yes | Not reported | Small | Yes | No | Yes | Authors state a small sample that limited their power. | 4/7 |
| Barry et al. (2011)[39] | Yes | Yes | Not reported | Unclear | Yes | No | Yes | No. | 4/7 |
| Beshgetoor et al. (2000)[41] | Yes | Yes | Not reported | Unclear | Yes | Cannot tell | Yes | No | 4/7 |
| Brown et al. (2000)[49] | Yes | Yes | 34/90 | Unclear | Yes | Yes | Yes | No | 5/7 |
| Campion et al. (2010)[34] | Yes | Yes | Cannot tell | Cannot tell | Yes | Cannot tell | Yes | No | 4/7 |
| Duncan et al. (2002)[21] | Yes | Yes | Not reported | State- or national-level representatives | Yes | Yes | Yes | No | 5/7 |
| Duncan et al. (2002)[45] | Yes | Yes | Not reported | State- or national-level representatives | Yes | Yes | Yes | Subsample of volunteers from a bigger study. | 5/7 |
| Fiore et al. (1996)[36] | Yes | Yes | Cannot tell | Cannot tell | Yes | Cannot tell | Yes | No | 4/7 |
| Guillaume et al. (2012)[38] | Yes | Yes | Not reported | Unclear | Yes | No | Yes | No. | 4/7 |
| Heinonen et al. (1993)[19] | Yes | Yes | Cannot tell | Cannot tell | Yes | Cannot tell | Yes | No | 4/7 |
| Hinrichs et al. (2010)[35] | Yes | Yes | Not reported | Unclear | Yes | No | Yes | No | 4/7 |
| Maïmoun et al. (2003)[25] | Yes | Yes | Not reported | Unclear | Yes | Cannot tell | Yes | No | 4/7 |
| Maïmoun et al. (2004)[24] | Yes | Yes | Not reported | Unclear | Yes | No | Yes | No | 4/7 |
| Medelli et al. (2009)[29] | Yes | Yes | Cannot tell | Cannot tell | Yes | Cannot tell | Yes | No | 4/7 |
| Medelli et al. (2009)[4] | Yes | Yes | Cannot tell | Cannot tell | Yes | Cannot tell | Yes | No | 4/7 |
| Morel et al. (2001)[27] | Yes | Yes | Not reported | Yes | Yes | No | Yes | Athletes who are successful and best adapted. | 5/7 |
| Nevill et al. (2004)[23] | Yes | Yes | Not reported | Unclear | Yes | No | Yes | No | 4/7 |
| Nichols et al. (2003)[32] | Yes | Yes | Not reported | Small | Yes | No | Yes | Authors stated limitations on sample size. | 4/7 |
| Nichols et al. (2010)[42] | Yes | Yes | Not reported | The control but not the cyclists. | Yes | No | Yes | Authors stated limited sample size and behavioral changes. | 4/7 |
| Nikander et al. (2005)[26] | Yes | Yes | Not reported | Unclear | Yes | No | Yes | No | 4/7 |
| Olmedillas et al. (2011)[40] | Yes | Yes | Not reported | Unclear | Yes | Yes | Yes | No | 5/7 |
| Penteado et al. (2001)[22] | Yes | Yes | No | Unclear | Yes | No | Yes | No | 4/7 |
| Rector et al. (2008)[37] | Yes | Yes | Not reported | Unclear | Yes | No | Yes | No | 4/7 |
| Rico et al. (1993)[20] | Yes | Yes | Cannot tell | Unclear | Yes | Cannot tell | Yes | No | 4/7 |
| Rico et al. (1993)[50] | Yes | Yes | Cannot tell | Unclear | Yes | Cannot tell | Yes | No | 4/7 |
| Sabo et al. (1996)[30] | Yes | Yes | Cannot tell | Unclear | Yes | Cannot tell | Yes | No | 4/7 |
| Stewart et al. (2000)[31] | Yes | Yes | Not reported | Unclear | Yes | Yes | Yes | No | 5/7 |
| Smathers et al. (2009)[33] | Yes | Yes | Not reported | Unclear | Yes | No | Yes | Cyclists who perform weight lifting were included | 4/7 |
| Warner et al. (2002)[28] | Yes | Yes | Not reported | Unclear | Yes | Yes | Yes | No | 5/7 |
| Wilks et al. (2009)[46] | Yes | Yes | Not reported | Unclear | Yes | Yes | Yes | No | 5/7 |
